# Evaluation of right ventricular longitudinal strain in pediatric patients with pulmonary hypertension by two-dimensional speckle-tracking echocardiography

**DOI:** 10.3389/fped.2023.1189373

**Published:** 2023-09-15

**Authors:** Qianjun Liu, Yuan Hu, Wenjuan Chen, Taoyue Yao, Wenfeng Li, Zhenghui Xiao, Jinqiao Liu, Yunbin Xiao

**Affiliations:** ^1^ Department of Ultrasound, Hunan Children’s Hospital, Changsha, China; ^2^ Intensive Care Unit, Hunan Children’s Hospital, Changsha, China; ^3^ Department of Cardiology, Hunan Children’s Hospital, Changsha, China

**Keywords:** pulmonary hypertension, pediatric, right ventricular strain, two-dimensional speckle tracking, echocardiography

## Abstract

**Objectives:**

We aimed to investigate the association between right ventricular longitudinal strain measured by two-dimensional speckle-tracking echocardiography (2D-STE) and right heart catheterization data in pediatric patients with pulmonary hypertension (PH).

**Methods:**

Two groups were evaluated, each consisting of 58 patients. Group 1, patients with PH; Group 2, normal matched controls. Data were collected from 58 patients with PH who underwent invasive hemodynamic evaluation. Standard transthoracic echocardiographic assessment was performed in all patients under the same circumstances. All patients underwent 2D-STE, and off-line analysis generated right ventricle longitudinal strain (RVLS) and right ventricular free wall strain (RVFW) and collected echocardiographic conventional parameters of right ventricular function, including the control group. The relationship between invasive characteristics and right ventricular function parameters was analyzed.

**Results:**

In all, 58 PH patients were included in our study. The mean pulmonary artery pressure (mPAP) and pulmonary vascular resistance (PVR) were strongly correlated with right ventricular free wall strain (RVFW) and right ventricular longitudinal strain (RVLS), moderately correlated with the right ventricle myocardial performance index (Tei index), weakly correlated with the transverse diameter of the right ventricle (RV) and the transverse diameter of the right atrium (RA), and moderately negatively correlated with right ventricular fractional area change (RVFAC). In terms of segments of the right ventricular free wall, the basal segment had the highest correlation coefficient with mPAP and PVR (*r* = 0.413, 0.523, 0.578, *r* = 0.421, 0.533, 0.575, *p *< 0.05, respectively). Tricuspid annular plane systolic excursion (TAPSE), main pulmonary artery diameter (MPA), peak systolic velocity of the right ventricle (RV-S’), and RA area parameters were not associated with mPAP and PVR (*p *> 0.05).

**Conclusions:**

Right ventricular longitudinal strain is a reliable indicator to evaluate right ventricular function in pediatric patients with PH. It can provide valuable reference information for the clinical judgment of the status and severity of the disease in children.

## Introduction

Assessing cardiac function in children is essential to clinical research, particularly in those with pulmonary hypertension (PH). To maintain diagnostic coherence and continuity in the management of pulmonary hypertension, treatment and follow-up from childhood to adulthood are recommended using the most recent guidelines (1). Although various complications have been reported, right heart failure is widely recognized as a serious consequence of PH; therefore, it is important to effectively evaluate right ventricular function in patients with PH. Echocardiography is still the most common method to evaluate cardiac function in the clinical setting. Due to the complex geometry of the right ventricle and the right ventricle functional parameters' dependence on stress, traditional echocardiographic parameters, such as right ventricular fractional area change (RVFAC) and tricuspid annulus plane systolic displacement (TAPSE), have limitations in predicting the prognosis of patients with PH (2). Two-dimensional speckle-tracking echocardiography (2D-STE) is used to quantitatively evaluate the degree and speed of myocardial deformation from different directions by tracking changes in myocardial motion. It is not angle-dependent and has been proven to have a good correlation with the results of cardiac function measured by cardiac magnetic resonance (CMR) (3). 2D-STE can analyze myocardial deformation by tracking myocardial motion and has been used to evaluate global and regional myocardial motion more effectively. Compared with traditional echocardiography, 2D-STE can, theoretically, reflect ventricular systolic and diastolic motion more comprehensively (4, 5). In recent years, STE has been used to evaluate left ventricular function in children with heart disease (6). Previous studies have confirmed that the measurement of left ventricular longitudinal strain (LVLS) by 2D-STE is a reliable quantitative descriptor for left cardiac function in children and is even better than left ventricular ejection fraction (EF) (7–10). Because of the irregular shape of the right ventricle, the evaluation of right ventricular function has always been difficult. Right ventricular myocardial fibers are mainly arranged longitudinally, and longitudinal motor function accounts for 75% of right ventricular systolic function (11). Therefore, analysis of right ventricular longitudinal motor function can represent right ventricular function (12). The application of right ventricular strain in adult studies has been extensively reported (13–15). Whether right ventricular strain can effectively and reliably evaluate right ventricular function in pediatric PH is the main purpose of this study. We analyzed the correlation between RV strain and right heart catheter data to verify the reliability of RV strain in evaluating right heart function in patients with PH and whether it is better than other traditional indicators.

## Methods

Pediatric PH patients were analyzed retrospectively from January 2021 to October 2022 at Hunan Children's Hospital. The PH group included patients with a mean pulmonary artery pressure (mPAP) > 20 mmHg when measured by catheterization and excluded those who previously suffered myocardial infarction, left heart failure (16), arrhythmia, and severe valvular disease, and those who were inconsolable and uncooperative. The control group included 58 age-matched healthy subjects, and the quality of all the images of the right ventricle met the standard for analysis. None of the patients had tricuspid or pulmonary valve stenosis. Written informed consent was obtained from the individual(s) or the minor(s)' legal guardian/next of kin for the publication of any potentially identifiable data included in this article.

**Figure 1:**
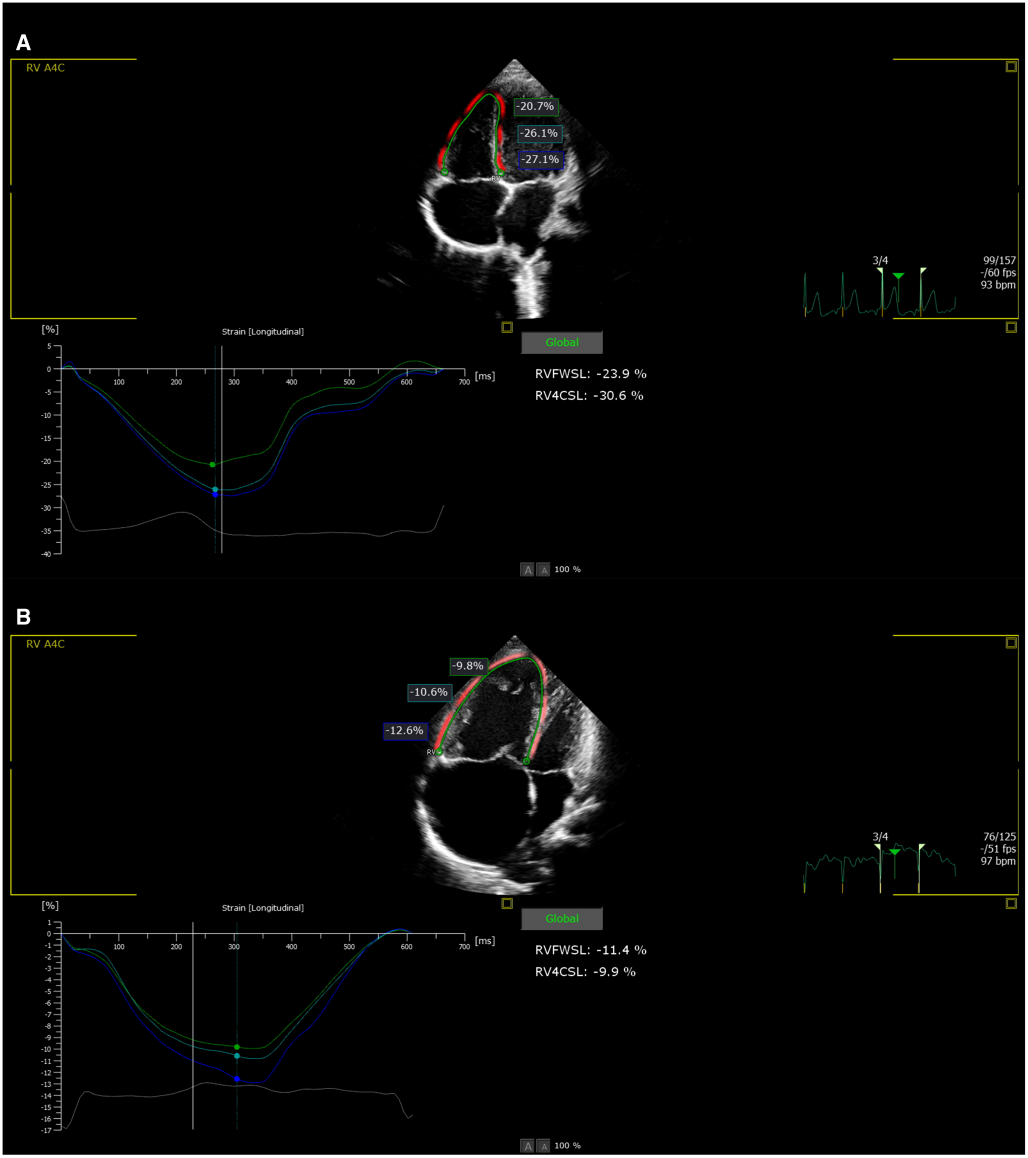
(**A**) Right ventricular strain value in normal group. (**B**) Right ventricular strain value in PH group.

The patients underwent echocardiograms that were fitted with 3–8 MHz and 1–5 MHz transducers (Philips EPIQ 7C systems). All studies were performed with simultaneous electrocardiographic monitoring. Four dynamic cardiac cycles (frame rate >40 fps) were stored in the supine or left decubitus position for postprocessing analysis. The analysis used Philips QLAB13 Autostrain RV analysis software; each index was measured three times and the average value was taken. 2D-STE was started on the four-chamber view and produced high-quality images, and the right ventricular myocardium was delineated along the endocardial plane. The delineation width and nodes were manually adjusted to obtain the right ventricular longitudinal strain (RVLS), right ventricular free wall strain (RVFW), right ventricular free wall apical strain (RVFW apic), right ventricular free wall midventricular strain (RVFW mid), and right ventricular free wall basal strain (RVFW bas) (Figure 1). Negative strain values indicate tissue shortening, and a smaller value (that is, a higher absolute value) indicates better RV systolic function. When a segment is not fully tracked, this set of data will not be included in the statistics. Right cardiac indices of pulmonary artery main internal diameter (MPA), transverse diameter of the right ventricle (RV), transverse diameter of the right atrium (RA), tricuspid annular plane systolic excursion (TAPSE), right ventricular fractional area change (RVFAC), right ventricle myocardial performance index (Tei index), RV peak systolic velocity (RV-S’) with Doppler tissue imaging (DTI) and area of the right atrium (RA area) were measured and collected.

The clearest tricuspid regurgitation image with the highest peak velocity was obtained, the velocity-time integral was recorded to obtain the average pressure gradient between the right ventricle and right atrium, and the mPAP was obtained by adding the right atrial pressure (RAP) (17). If tricuspid regurgitation could not be clearly obtained, the mPAP was estimated by measuring the early velocity of pulmonary regurgitation (18). Estimation of the RAP is based on the inferior vena cava width and the respiratory change rate (19). Images were collected and measurements were taken by two experienced pediatric cardiologists. A dataset of twenty children was randomly selected for the intraobserver and interobserver analysis to observe any variation in the measured echocardiographic indicators.

### Right cardiac catheterization

The Swan-Ganz floating catheter was inserted percutaneously into the pulmonary arterioli from the right femoris vein through the right atrium, right ventricle, and pulmonary artery to continuously monitor pressure changes such as the mPAP and the pulmonary artery wedge pressure (PAWP), and cardiac output (CO) was measured by Fick's method. The pulmonary resistance (pulmonary vascular resistance, PVR) index was calculated. The PVR in the control group was determined by the integration of peak tricuspid regurgitation velocity/flow velocity of the right ventricular outflow tract (20). The time interval between ultrasound and catheter was within 24 hours.

### Reproducibility

Twenty participants were selected from the normal control group randomly, and the 2D-STE was measured again by the same surveyor and then measured independently by another surveyor.

The data were analyzed using SPSS version 22.0 and MedCalc version 19.7. Summary data are expressed as the mean values ± standard deviations or percentage of patients. The independent sample *T*-test was used to compare the two groups of samples for the ultrasound parameters and right cardiac catheter data. The Pearson correlation method was used to analyze the correlation between the right cardiac catheter data and ultrasound parameters. Intraobserver and interobserver agreement was based on a combination of Bland–Altman plots. A *p*-value <0.05 was considered statistically significant.

## Results

### Study population

A total of 58 pediatric patients with PH were enrolled in the study, including 30 boys and 28 girls. The ages of the patients in the study group ranged from 3 years 4 months to 8 years 6 months, with an average age of 6 ± 2.6. There were 38 cases of congenital heart disease combined with pulmonary hypertension (CHD-PH), 8 cases of idiopathic pulmonary arterial hypertension (IPAH), and 12 cases of bronchopulmonary dysplasia with pulmonary hypertension (BPD-PH). A total of 58 healthy volunteers were enrolled in the study. The ages of the patients in the control group ranged from 2 years and 9 months to 8 years and 7 months, with an average age of 5.8 ± 2.9. The baseline clinical and demographic characteristics of all the patients and the individual PH and normal groups are listed in Table 1.

**Table 1:** Baseline characteristics.

Characteristic	Normal (*n* = 58)	PH (*n* = 58)	*p*
Gender female/male	28/38	28/38	NA
Age, years	5.8 ± 2.9	6 ± 2.6	0.368
Height, cm	125 ± 29.65	104.26 ± 25.27	<0.05
Weight, kg	22.8 ± 14.6	17.27 ± 8.66	0.248
Heart rate, /min	89 ± 5	86 ± 6	<0.05
BSA	0.90 ± 0.32	0.70 ± 0.11	0.24
SBP, mmHg	119 ± 14.6	115 ± 13.9	<0.05
DBP, mmHg	63.7 ± 12.58	65.3 ± 11.98	<0.05
CHD-PH	0	38	NA
IPAH	0	8	NA
BPD-PH	0	12	NA

Data are expressed as mean ± SD.

BSA, body surface area; SBP, systolic blood pressure; DBP, diastolic blood pressure; CHD-PH, congenital heart disease combined with pulmonary hypertension; IPAH, idiopathic pulmonary arterial hypertension; BPD-PH, bronchopulmonary dysplasia with pulmonary hypertension.

### Comparison of ultrasonic indicators between the PH group and the control group

Compared with those in the control group, the RV, RA, RA area, MPA, mPAP, and PVR increased, while RVFAC, RV-S', Tei index, TAPSE, absolute values of RVLS and RVFW, and absolute values of right ventricular free wall segments decreased in the PH group. The differences were statistically significant (*p *< 0.05) (Table 2).

**Table 2:** Comparison of right ventricular parameters between normal and PH group.

Groups	Normal	PH	*p*
*N*	58	58	NA
MPA (mm)	13.85 ± 4.34	22.19 ± 7.12	0.002
RV (mm)	20.64 ± 6.69	32.72 ± 11.22	<0.001
RA (mm)	26.19 ± 5.77	38.26 ± 16.2	0.003
RA area (cm^2^)	8.18 ± 2.95	13.06 ± 11.34	0.006
TAPSE (mm)	15.38 ± 4.79	12.00 ± 3.97	0.018
RVFAC (%)	62.74 ± 5.36	33.64 ± 14.38	<0.001
Tei index	0.41 ± 0.08	0.63 ± 0.23	0.024
RV-S' (cm/s)	13.65 ± 2.29	9.32 ± 2.48	0.012
RVLS (%)	−25.99 ± 2.26	−12.73 ± 5.29	<0.001
RVFW (%)	−27.14 ± 2.22	−10.76 ± 7.69	<0.001
RVFW apic (%)	−24.41 ± 2.16	−12.02 ± 6.25	<0.001
RVFW mid (%)	−27.08 ± 2.63	−14.14 ± 6.47	<0.001
RVFW bas (%)	−29.92 ± 3.66	−15.42 ± 7.16	<0.001
mPAP (mmHg)	23.08 ± 5.67	45.34 ± 12.53	<0.001
PVR (Wood)	1.5 ± 0.76	12.56 ± 6.45	<0.001

Data are presented as mean ± SD. Normal group’s mPAP was measured by tricuspid regurgitation. PVR in control group was determined by the integration of peak tricuspid regurgitation velocity/flow velocity of right ventricular outflow tract.

MPA, main pulmonary artery diameter; RV, transverse diameter of right ventricle; RA, transverse diameter of right atrial; TAPSE, tricuspid annular plane systolic excursion; RVFAC, right ventricular fractional area change; RV-S’, the peak systolic velocity of right ventricle; RVLS, right ventricular longitudinal strain; RVFW, right ventricular free wall strain; RVFW apic, right ventricular free wall apical strain; RVFW mid, right ventricular free wall midventricular strain; RVFW bas, right ventricular free wall basal strain; mPAP, mean pulmonary artery pressure; PVR, pulmonary vascular resistance.

### Correlation between invasive characteristics and ultrasound indicators

The mPAP and PVR were strongly correlated (0.6–0.8) with RVFW and RVLS, moderately correlated (0.4–0.6) with the Tei index, and weakly correlated (0.2–0.4) with RV and RA, and moderately negatively correlated with RVFAC. Regarding segments of the right ventricular free wall, the basal segment had the highest correlation coefficient with mPAP and PVR (*r* = 0.413, 0.523, 0.578, *r* = 0.421, 0.533, 0.575, *p *< 0.05, respectively). TAPSE, MPA, RV-S', and RA area parameters were not associated with mPAP and PVR (*p *> 0.05) (Table 3).

**Table 3:** Correlation analysis between invasive characteristics with echocardiographic indicators.

Parameter	mPAP		PVR	
Echocardiographic data	*r*	*p*	*r*	*p*
RVFW (%)	0.656	<0.05	0.638	<0.05
RVLS (%)	0.628	<0.05	0.617	<0.05
RVFW apic (%)	0.413	<0.05	0.421	<0.05
RVFW mid (%)	0.523	<0.05	0.533	<0.05
RVFW bas (%)	0.578	<0.05	0.575	<0.05
RVFAC (%)	−0.585	<0.05	−0.563	<0.05
Tei index	0.529	<0.05	0.443	<0.05
RV (mm)	0.374	<0.05	0.312	<0.05
RA (mm)	0.342	<0.05	0.302	<0.05
RV-S’ (cm/s)	−0.305	0.066	−0.294	0.059
TAPSE (mm)	−0.232	0.167	−0.212	0.123
RA area (cm^2^)	0.223	0.255	0.234	0.289
MPA (mm)	0.163	0.323	0.123	0.356

PVR, pulmonary vascular resistance; mPAP, mean pulmonary artery pressure; RVLS, right ventricular longitudinal strain; RVFW, right ventricular free wall strain; RVFW apic, right ventricular free wall apical strain; RVFW mid, right ventricular free wall midventricular strain; RVFW bas, right ventricular free wall basal strain; RVFAC, right ventricular fractional area change; RV, transverse diameter of right ventricle; RA, transverse diameter of right atrial; RV-S’, the peak systolic velocity of right ventricle; TAPSE, tricuspid annular plane systolic excursion; MPA, main pulmonary artery diameter.

Interestingly, 2 cases of severe right heart failure were found during the statistical analysis. Their FAC and absolute values of RVLS and RVFW were significantly reduced and PVR was increased, while the mPAP indicated mild pulmonary hypertension pressure.

### Reproducibility

STE had high repeatability and consistency in measuring right ventricular strain in the normal group. The 2D-STE was used by the same surveyor for the second time and the intraobserver agreement as measured by the ICC was very good (Table 4). The second surveyor measured the above 20 children again and the interobserver agreement as measured by the ICC was very good (Table 4). The intra-observer and inter-observer Bland–Altman plots showed that the 95% confidence intervals were clinically acceptable (Supplementary Figure 1 and Supplementary Figure 2).

**Table 4:** Inter-and intra-observer analysis.

Measurements (%)	Inter-observer	ICC Inter-observer	*P*-value Inter-observer	Intra-observer	ICC Intra-observer	*P*-value Intra-observer
RVLS	25.57 ± 1.79	0.915	<0.01	25.57 ± 1.79	0.920	<0.01
	25.72 ± 1.55			25.94 ± 1.61		
RVFW	26.53 ± 1.05	0.846	<0.01	26.53 ± 1.05	0.837	<0.01
	26.73 ± 0.86			26.80 ± 1.05		
RVFW apic	24.38 ± 1.50	0.893	<0.01	24.38 ± 1.50	0.853	<0.01
	24.63 ± 1.57			24.61 ± 1.47		
RVFW mid	26.34 ± 1.93	0.912	<0.01	26.34 ± 1.93	0.837	<0.01
	26.46 ± 1.47			26.54 ± 1.61		
RVFW bas	28.88 ± 1.36	0.836	<0.01	28.88 ± 1.36	0.840	<0.01
	29.10 ± 1.26			29.24 ± 1.42		

Data are presented as mean ± SD.

InterCC, Interclass correlation coeffificient; RVLS, right ventricular longitudinal strain; RVFW, right ventricular free wall strain. RVFW apic, right ventricular free wall apical strain; RVFW mid, right ventricular free wall midventricular strain; RVFW bas, right ventricular free wall basal strain.

## Discussion

We applied 2D-STE to evaluate right ventricular function in pediatric patients with PH. The absolute values of RVLS and RVFW were decreased in the PH group and had significantly correlated coefficients with the mPAP and the PVR when compared with other indicators. The results showed that strain was more sensitive to changes in right heart pressure than other indicators. The absolute value of right ventricular strain decreased with increasing pulmonary artery pressure and pulmonary vascular resistance. The decrease in the absolute value of right ventricular strain also indicates deterioration of right ventricular systolic function. Reduced systolic function of the right ventricle leads to decreased output of the right ventricle, which further increases the volume load. Long-term right ventricular afterload and end-diastolic volume increase with pulmonary hypertension, leading to increased myocardial cell length, reintegration of sarcomeres, right ventricular compensatory remodeling (21), right ventricular wall hypertrophy, and severe increases in right ventricular load, which further inhibit right ventricular contraction. In particular, the extensive proliferation of subendocardial interstitial fibers and perivascular fibers (22) also reduced the motion amplitude of the ventricular wall and affected the strain of the right ventricle. This vicious cycle leads to right heart failure. Therefore, the adaptation of right ventricular function to increased afterload, known as RV-arterial coupling, is key to determining the prognosis of pulmonary hypertension (23), so the treatment strategy cannot be determined by pulmonary artery pressure alone. Pulmonary artery pressure is determined by cardiac output and PVR. When right ventricular function decreases significantly, right cardiac output decreases, and pulmonary artery pressure may not increase even though pulmonary vascular resistance increases with disease progression. Increased PVR is the result of pathological changes in the pulmonary artery, resulting in RV pressure overload and RV systolic dysfunction in PH. RV pressure overload directly affects RV longitudinal systolic function and results in impaired RVLS. PH is the result of an abnormal mismatch between the right ventricle and pulmonary vessels, which reminds us that clinicians should pay more attention to right ventricular function than to the level of pulmonary artery pressure.

In RV strain analysis, the longitudinal strain was divided into RVLS and RVFW. The RVLS value includes the strain value of the ventricular septum and RVFW. In our study, the correlation coefficient of RVFW with the mPAP and the PVR was higher than that of RVLS, considering that RVLS is affected by the ventricular septum. Because it also participates in the systolic and diastolic movements of the left ventricle, the free wall fully acts on the right ventricle, which can better reflect the function of the right ventricle (24, 25). Among the three segments of the right ventricular free wall, the basal segment had the highest correlation coefficient with the mPAP and the PVR, indicating that the basal segment of the right ventricular free wall played an important role in right ventricular contraction, while the apical segment played a lesser role. In fact, our study had 2 cases of severe dyskinesia in the late stage of PH, and the absolute value of right ventricular strain was significantly decreased, but the assessed pulmonary artery pressure indicated mild pulmonary hypertension, which indicated that the right ventricular strain had high sensitivity in evaluating the decrease in right ventricular function, which was consistent with the patient's condition (11). A meta-analysis also showed that RVLS can independently predict the prognosis of PH and may even be of value in optimizing the current predictive model to better predict clinical events or mortality in PH patients (13). Interobserver and intraobserver agreement was measured by the interclass correlation coefficient (ICC) and showed a strong correlation for all measurements. It was confirmed that right ventricular strain measurement has high repeatability and reliability.

### Limitations

The sample size was small, and the follow-up time was not long enough. We cannot assess whether RVLS provides long-term prognostic information. Only a few types of pulmonary hypertension diseases were included, and the effects of different causes on right ventricular systolic function may be different. We analyzed the RVLS with stored images; however, its utility may be influenced by image quality and different echocardiography machines. Second, we used a vendor-independent platform, and it may be more rigorous to measure RVLS using echocardiographic images studied by different echocardiographic machines. Multicenter long-term follow-up studies with larger sample sizes are needed in the future to verify the reliability of the conclusions of this study.

## Conclusion

Right ventricular longitudinal strain is a reliable indicator for evaluating right ventricular function in pediatric patients with PH and can provide valuable reference information for the clinical diagnosis and treatment of PH. We recommend it as a routine indicator to evaluate cardiac function in pediatric patients.

## Data Availability

The original contributions presented in the study are included in the article/**Supplementary Material**, further inquiries can be directed to the corresponding authors.

## Supplementary material

Supplementary Figure 1 Bland-Altman plot of intraobserver measurements of RVLS, RVFW, RVFW apic, RVFW mid, RVFW bas. (**A**) RVLS, right ventricular longitudinal strain; (**B**) RVFW, right ventricular free wall strain; (**C**) RVFW apic, right ventricular free wall apical strain; (**D**) RVFW mid, right ventricular free wall midventricular strain; (**E**) RVFW bas, right ventricular free wall basal strain. *N* = 20.

Supplementary Figure 2 Bland-Altman plot of interobserver measurements of RVLS, RVFW, RVFW apic, RVFW mid, RVFW bas. (**A**) RVLS, right ventricular longitudinal strain; (**B**) RVFW, right ventricular free wall strain; (**C**) RVFW apic, right ventricular free wall apical strain; (**D**) RVFW mid, right ventricular free wall midventricular strain; (**E**) RVFW bas, right ventricular free wall basal strain. *N* = 20.
