# Supplementary figures and images for: Evaluation of right ventricular longitudinal strain in pediatric patients with pulmonary hypertension by two-dimensional speckle-tracking echocardiography
Source: Front Pediatr. 2023 Sep 15;11:1189373. doi: 10.3389/fped.2023.1189373 (PMC10540637; doi:10.3389/fped.2023.1189373)

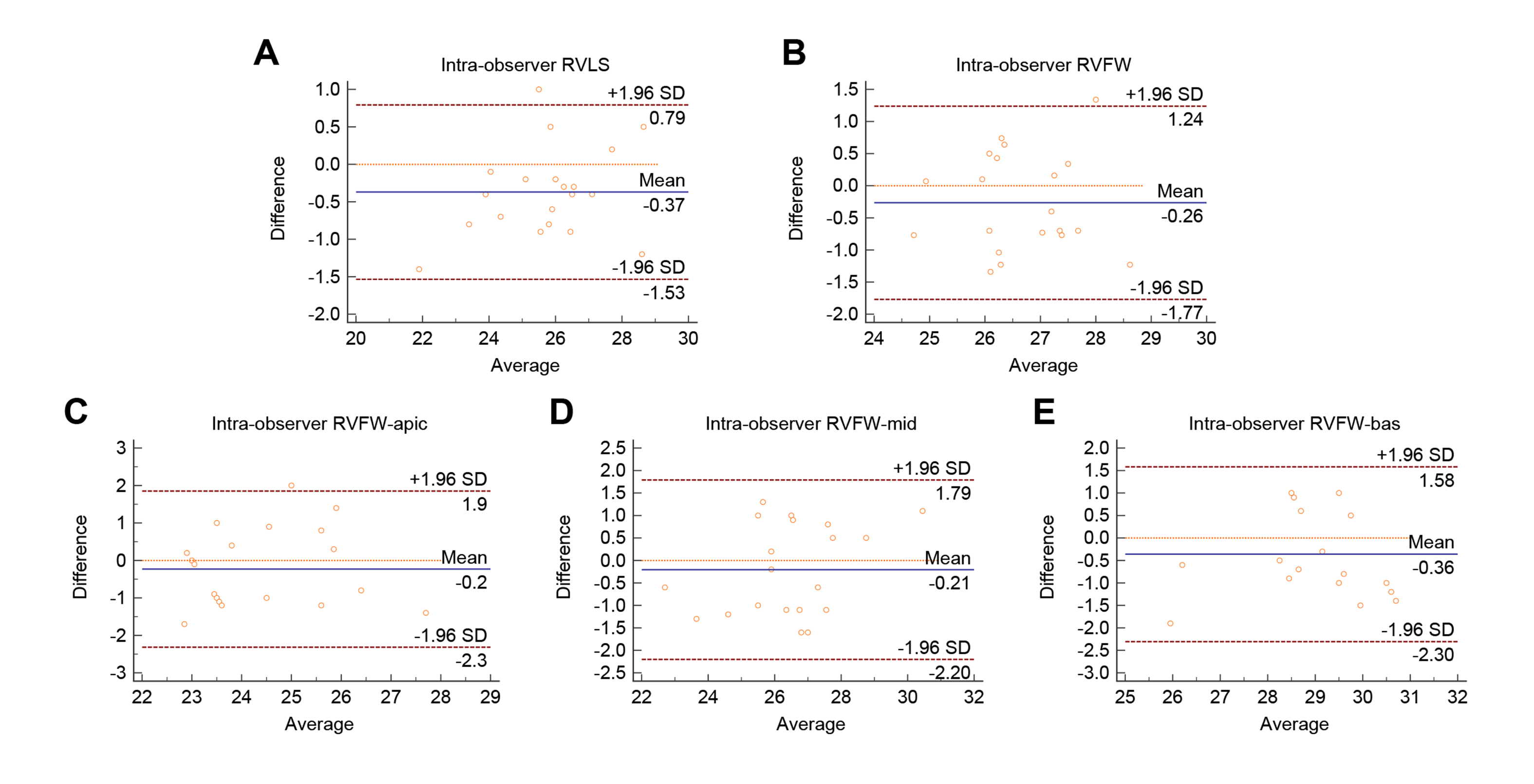

Supplement: Supplementary Figure 1 — Bland-Altman plot of intraobserver measurements of RVLS, RVFW, RVFW apic, RVFW mid, RVFW bas. (A) RVLS, right ventricular longitudinal strain; (B) RVFW, right ventricular free wall strain; (C) RVFW apic, right ventricular free wall apical strain; (D) RVFW mid, right ventricular free wall midventricular strain; (E) RVFW bas, right ventricular free wall basal strain. N = 20. [file Image1.tif]

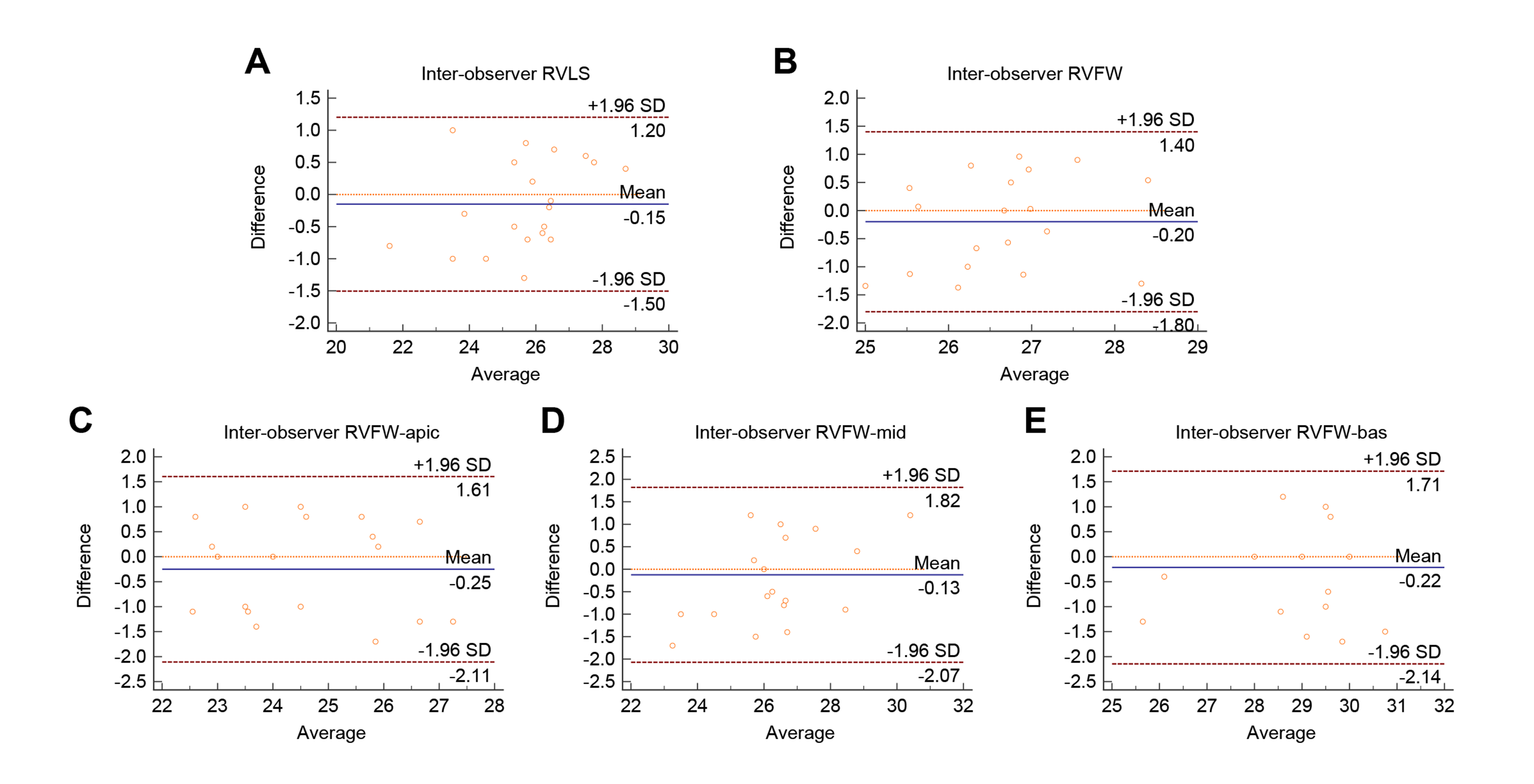

Supplement: Supplementary Figure 2 — Bland-Altman plot of interobserver measurements of RVLS, RVFW, RVFW apic, RVFW mid, RVFW bas. (A) RVLS, right ventricular longitudinal strain; (B) RVFW, right ventricular free wall strain; (C) RVFW apic, right ventricular free wall apical strain; (D) RVFW mid, right ventricular free wall midventricular strain; (E) RVFW bas, right ventricular free wall basal strain. N = 20. [file Image2.tif]
